# Development of a social distancing monitoring system in Republic of Korea: results of a modified Delphi process

**DOI:** 10.1186/s12889-022-13277-8

**Published:** 2022-04-29

**Authors:** Suin Seo, Jin-Ok Han, Sool Shin, Heeyoung Lee

**Affiliations:** 1 Gyeonggi Public Health Policy Institute, 7th floor, 172, Dolma-ro, Seongnam-si, Gyeonggi-do 13605 Republic of Korea; 2 Seoul National University Bundang Hospital, 82, Gumi-ro 173 Beon-gil, Seongnam-si, Gyeonggi-do 13620 Republic of Korea

**Keywords:** Social distancing, Monitoring, System, Modified Delphi process, South Korea

## Abstract

**Background:**

Social distancing policies work in different ways and at different levels. In addition, various forms of monitoring systems have been implemented in different countries. However, there is an almost complete lack of specific monitoring system in Republic of Korea to effectively monitor social distancing measures compliance and outcome. This study aims to develop a monitoring system for social distancing measures compliance and outcome in Korea to evaluate and improve the implemented policy.

**Methods:**

A draft monitoring system was developed after reviewing Korea’s social distancing measures (central and local government briefings) and checking available data about social distancing behavior. The modified Delphi process was used to evaluate the draft of the social distancing monitoring system. In total, 27 experts participated in the evaluation. The round 1 evaluation includes (1) commenting on the composition of the monitoring fields (open response), (2) monitoring indicators for each monitoring field (10-point Likert scale), and (3) commenting on the source of data used to develop the monitoring system (open response). In the round 2 evaluation, 55 indicators, excepting open responses, were re-evaluated.

**Results:**

The response rate for the Delphi survey was 100% in both the first and second rounds. Of the 55 indicators, 1 indicator, which did not satisfy the quantitative criteria, was excluded. According to the experts’ open response comments, 15 indicators were excluded, as these indicators overlapped with other indicators or had little relevance to social distancing. Instead, 23 new indicators were added. Finally, 62 indicators were included with 12 available data sources. The monitoring system domain was divided into ‘social distancing measures state, social distancing measures compliance, social distancing outcome’.

**Conclusions:**

This study is significant in that it is the first in Korea to develop a comprehensive monitoring system for checking if social distancing measures are being followed well, and is applicable to estimates utilizing data that are immediately available for each indicator. Furthermore, the developed monitoring system could be a reference for other countries that require the development of such systems to monitor social distancing.

**Supplementary Information:**

The online version contains supplementary material available at 10.1186/s12889-022-13277-8.

## Background

On March 11, 2020, the World Health Organization (WHO) declared the COVID-19 pandemic. In total, more than 118,300 people had been infected, and 4291 had died in 114 countries [1]. To overcome the situation, several pharmaceutical companies have been working to develop vaccines and treatments. Vaccination is taking place worldwide. However, the effects of vaccination for preventing the spread of viruses will take some time and Covid-19 virus variants continue to be reported [2]; thus, public health measures such as wearing masks and maintaining social distance would likely remain important [3, 4].

Social distancing is a public health measure that reduces the spread of viruses by reducing physical contact between people [5]. Social distancing measures are defined and implemented in various ways at different levels depending on the country [6]. The WHO has delineated guidelines for social distancing measures according to situations and locations (e.g., workplaces, schools, group gatherings, public places, and transportation) [7]. The European Centre for Display Prevention and Control (ECDC) defined and delineated social distancing according to the individual and group level [8].

As social distancing policies vary, various monitoring systems are available [9–11]. Social distancing worldwide is mainly monitored by non-profit organizations and understood as the presence or degree of implementation of the policy in different countries. In addition, various private organizations such as Google provide data to identify social distancing levels [12–14]. While some institutions presented “blocking and closing” and “healthcare systems” on a 100-point scale based on policy intensity [10], others evaluated whether implementing such affective measures would impact the socioeconomic environment, healthcare system capabilities, and government intervention [9]. A few studies have proven the efficacy of social distancing policy in preventing the spread of COVID-19 beyond monitoring [5, 6].

In Republic of Korea, several institutions have attempted to analyze and present only part of the social distancing behavior [15, 16]. The National Disaster and Safety Status Control Center has announced the results of analyzing mobile phone traffic, credit card use data, and public transportation utilization in the Seoul metropolitan area. Private research companies also reported indicators based on their regular COVID-19 risk perception survey results such as individuals’ social distancing practice rate, and restrictions on outdoor activities or eating out. However, the above results have a limitation that only a part of social distancing behavior was analyzed and it was only a one-time analysis. Therefore, social distancing measures compliance and outcome have not yet been sufficiently evaluated and no comprehensive monitoring system is in place.

This study aims to develop an appropriate monitoring system of social distancing measures compliance/outcome and indicators based on available data sources, identify the status of social distancing measures and behavior in Korea, and utilize them for policy evaluation and improvement in the future.

## Methods

### Study design

**Fig. 1:**
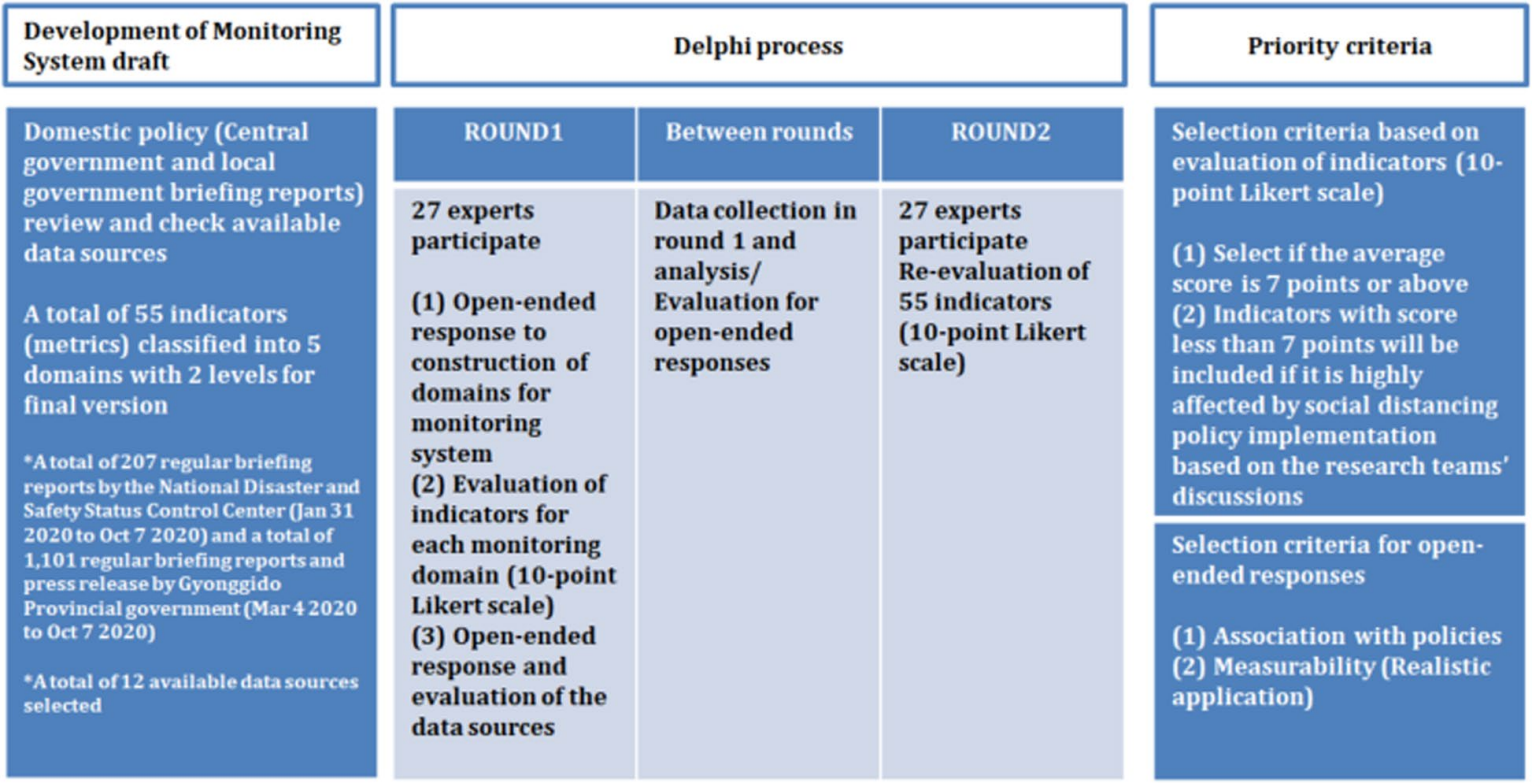
Methodological workflow

The modified Delphi process [17], consisting of two surveys and a three-step process, was used to reach consensus on the overall draft of the developed monitoring system for social distancing domains and indicators. The Delphi method is used to achieve consensus on a particular topic by having experts vote through multiple meetings and provide feedback [18, 19]. The difference between the modified and conventional Delphi process is that it can reduce the number of surveys from rounds 3 to 2 using structured questionnaires from the beginning, improving the convenience and efficiency of the survey. In addition, it was carried out online to increase the participation of experts from various fields and overcome the shortcoming of converging expert opinions according to their reputations and authorities. After evaluating the primary draft (checking scores and open responses) in the first round, we obtained consensus on open responses through adding and deleting indicators. In the second round, we reassessed the drafts (score checking only) to establish a final monitoring system. Figure 1 shows a flowchart of the study method.

### Participant recruitment

Since social distancing is a policy [20] to prevent the spread of COVID-19, 27 experts were recruited based on two major conditions (Table 1). They were (1) 15 clinical healthcare professionals related to COVID-19(11 department of infectious diseases, 1 pulmonology, 1 psychiatry, 1 pediatrics, 1 emergency medicine), and (2) 12 healthcare system development and analysis specialists (7 preventive medicine, 2 social medicine, 2 occupation & environment Medicine, 1 health communication). The composition of experts included not only those knowledgeable in the COVID-19 virus, but also other infectious disease specialists, nursing professionals who work with routine infection control and patient management in hospitals, and public health and preventive medicine specialists with experience and knowledge in public health measures and policies. The majority of experts are policy-makers on public health measures, including social distancing in Korea. They were 1 Health Care Committee (National government level), 7 Distancing in Daily Life Committee (National government level), 13 COVID-19 Emergency Response Committee (Local government levels) and 6 Policy Committee of infectious disease society of Korea (Academic). Experts were invited to participate in the modified Delphi process via email. Those who agreed to participate were sent an email with an online link to conduct the first survey.

**Table 1:** Delphi Panellists (*N* = 27)

	Number	Percentage
**Specialty**		
Clinical healthcare professionals related to COVID-19	15	56%
Healthcare system development and analysis specialists	12	44%
**Role and affiliation related to COVID-19 policy making**		
Health Care Committee (National government level)	1	4%
Distancing in Daily Life Committee (National government level)	7	26%
COVID-19 Emergency Response Committee (Local government levels)	13	48%
Policy Committee of infectious disease society of Korea (Academic)	6	22%

### Initial dimensions and criteria of monitoring system

Only a few studies have investigated social distancing policies and behavior related to COVID-19 [21–23]. In addition, while various forms of social distancing monitoring systems are being established in other countries, there are several limitations to the use of a monitoring system in Korea. First, since different countries have implemented social distancing measures in various ways, it is not appropriate to include their measures indicators (e.g., complete lockdowns, restrictions on movement between cities) that have not been considered in Korea. Second, even though similar measures may have been implemented in Korea, these are still difficult to apply because the recommended level or detailed methods may differ. Because of these problems, we believed that a systematic review of the methodology and monitoring systems in other countries would not be suitable for this study. Therefore, this study reviewed social distancing measures at the national and local government levels and available data sources about social distancing measures compliance and outcome to develop a monitoring system draft. First, 207 regular COVID-19 infection briefings (from January 31 to October 7, 2020) by the National Disaster and Safety Status Control Center were reviewed to understand the overall social distancing measures in Korea. In addition, 1101 regular press briefings and press releases issued (from March 4 to October 7, 2020) by the Gyeonggi-do provincial government were reviewed to determine whether a special form of social distancing measures was implemented [24, 25]. Domestically available data sources about social distancing compliance and outcome included both public and private data; thus, 12 data sources including secondary data from surveys were selected. Furthermore, 55 indicators were selected according to available data sources in Korea, and the monitoring system domain was divided into five categories based on two levels (Table 2).

### Data analysis

#### Round 1

The first round of the modified Delphi process was conducted for 6 days from July 9–14, 2020. In total, 27 experts from various fields received e-mails with information on the overall research, purpose of the modified Delphi process, detailed monitoring domains and indicators, and survey links for the appropriateness of the data source assessment. The survey consisted of (1) feedback on monitoring domains (open response), (2) evaluation of indicators by monitoring domain (10-point Likert scale: from “1=no need at all” to “10 = absolutely needed” and Open Responses), and (3) opinions regarding the data sources used (Open Response). Based on SMART criteria [26], each indicator was evaluated on a 10-point Likert scale so that quantitative results could be estimated, and the open responses were utilized to provide various opinions for the overall evaluation parts. The ‘SMART’ criteria (Specific, Measurable, Attributable, Reliable, and Timely) is often used in developing indicators for monitoring and evaluation. Those who had not yet completed the survey were reminded to do so 3 days before the deadline. The response rate for the first Delphi survey was 100%.

#### Between rounds

Data from round 1 were collected and analyzed. For the evaluation of the indicators, the mean, median, and standard deviations were calculated. SAS 9.3 was used for the statistical analysis. The second round of the survey was conducted for indicators with an average score of 7 or higher. All open responses were submitted to the entire research team prior to the initiation of the second round of the study, including recommendations for changes to other indicators or comments regarding the addition or exclusion of indicators, as well as to the purposes and scope of the monitoring domains or opinions about data sources. In response, our research team members discussed whether to reflect on the study based on accessibility, feasibility, timeliness, etc. Thus, except for the open response questions, the second round of evaluation for indicators in each monitoring domain was conducted using a 10-point Likert scale. In the second round, the results from the first round (average and median) were shared with the experts by providing the survey link via e-mail.

#### Round 2

The second round of the modified Delphi process lasted 6 days from July 17–22, 2020. The survey was written using Google Forms and distributed to the same experts (*n* = 27). An assessment of the same 55 indicators by monitoring domain as in the first round was performed in the second round. The mean and median values were provided for each indicator and evaluated on a 10-point Likert scale using the same SMART criteria as in the first round. Those who did not complete the survey were reminded to do so 1 day before the deadline. The response rate for the second Delphi survey was 100%.

### Priority

The most important principles for developing a monitoring system were (1) policy linkage and (2) measurability (application in reality), which were reflected in the open response comments. In addition, the indicators were chosen based on an average score of 7 or higher and included any other indicators from the discussion among research team members, which may have been greatly affected by the social distancing policy.

## Results

### Round 1 – based on monitoring indicator evaluations

In total, 55 potential indicators were included in the first round of voting (Table 2), and all indicators excepting “public transportation restrictions” (6.93 points), “residential mobility rate” (6.89 points), and “high expenses for daily necessities experience rate” (6.85 points) were evaluated as higher than 7 points. Thus, all indicators were evaluated in the second round. However, apart from the calculated scores, the exclusion, segmentation, and addition of indicators were suggested in the experts’ open responses. Additional discussions to reflect on their opinions were conducted. Open responses will be addressed in the discussion on these later in the paper.

**Table 2:** Results of the assessments of indicators in the Delphi process

Domain 1	Domain 2	Round 1			Round 2		
		Mean	Median	SD	Mean	Median	SD
**Direct-Policy implementation level**	1. Level of social distancing policy	9.30	10	1.61	9.50	10	1.07
	2. School closures	8.85	9	1.70	8.85	9	1.19
	3. Workplace closures	8.11	9	2.06	8.35	9	1.13
	4. Cancellation of events	8.33	9	1.71	8.54	8.5	1.07
	5. Restrictions on rallies/demonstrations	8.41	9	1.67	8.69	9	0.88
	6. Restrictions on the use of transportation	6.93	8	2.60	7.04	7.5	1.48
	7. Staying indoors (at home)	7.56	8	1.95	7.50	8	0.99
	8. Restrictions on domestic travel	7.11	7	2.19	6.92	7	1.20
	9. Control on international travel	8.52	9	1.72	8.77	9	0.86
**Direct-Community level**	10. Rate of implementation of telecommuting	8.41	8	1.39	8.23	8	0.91
	11. Experience rate of working even when sick	8.59	9	1.47	8.81	9	0.90
	12. Facility measures performance rate	8.15	9	2.14	8.35	8	1.09
	13. Floating population change	8.41	9	1.97	8.54	9	1.17
**Direct-individual level**	14. Facial mask wearing rate	9.52	10	0.80	9.69	10	0.55
	15. Indoor facial mask wearing rate	9.15	10	1.38	9.54	10	0.76
	16. Hand washing practice rate	9.26	10	1.10	9.54	10	0.71
	17. Use rate of hand sanitizers or soap	8.30	9	1.81	8.65	9	1.38
	18. Degree of daily life change	8.11	8	1.60	8.00	8	0.94
	19. Residential mobility rate	6.89	7	1.74	6.85	7	1.32
	20. Experience rate of the cancellation of hospital visits	7.59	8	1.87	7.27	7	1.19
	21. Movement rate in retail stores and leisure facilities	8.41	8	1.45	7.96	8	1.11
	22. Movement rate in grocery stores and pharmacies	7.96	8	1.68	7.62	8	1.13
	23. Movement rate in parks	7.37	7	1.90	7.12	7	1.42
	24. Restriction rate of using multi-use facilities	8.44	9	1.50	8.31	8	1.05
	25. Restriction rate of outdoor activities	7.93	8	1.52	7.77	8	0.99
	26. Restriction rate of eating out	7.74	8	1.40	7.62	8	0.98
	27. Experience rate of the cancellation of group gatherings (dinner)	7.67	8	1.44	7.69	8	0.84
	28. Restriction rate on the use of public transportation	7.67	8	1.69	7.77	8	0.76
	29. Change rate of movement at public transportation stops	7.37	8	1.94	7.46	8	1.39
	30. Change rate of bus use	7.89	8	1.74	7.92	8	1.02
	31. Change rate of subway use	7.89	8	1.74	7.81	8	1.20
	32. Change rate of taxi use	7.59	8	1.50	7.62	8	1.10
	33. Change rate of car use	7.96	8	1.40	8.00	8	0.94
	34. Change rate of railway use	7.44	8	1.80	7.65	8	1.20
	35. Purpose-specific transportation utilization rate	7.04	7	1.74	7.00	7	1.10
	36. Change rate of domestic flight use	7.63	8	1.84	7.73	8	1.28
	37. Experience of domestic travel	7.81	8	1.64	7.58	8	1.03
	38. Change rate of tourist visits at hotspots	8.15	8	1.54	7.92	8	0.98
	39. Change rate of international flight use	7.93	8	1.90	7.85	8	1.19
	40. Change rate of foreigner arrivals	8.22	9	1.86	8.19	8	1.27
	41. Change rate of resident (Korean) departures	8.19	8	1.80	8.00	8	1.30
	42. Rate of controlling working hours	7.85	8	1.58	7.69	8	1.01
	43. Commuting rate	7.07	7	1.74	6.88	7	1.24
**Indirect level**	44. Change rate of non-contact purchases	8.30	8	1.37	8.27	8	0.92
	45. Change rate of food delivery	8.41	8	0.92	8.27	8	0.53
	46. Change rate of TV home shopping, and Internet shopping purchases	8.11	8	1.58	7.96	8	0.77
	47. Experience rate of purchasing necessities in bulk	6.85	7	1.63	6.77	7	1.31
	48. Change rate in the number of restaurants/entertainment sales	8.04	8	1.32	7.88	8	0.91
	49. Change rate of private academy sales	7.33	8	1.69	7.27	8	1.31
	50. Change rate in the number of sports/culture/leisure facility sales	7.74	8	1.61	7.54	8	1.36
	51. Change rate in the number of travel/transportation sales	8.04	8	1.45	7.88	8	1.14
**Others**	52. Practice rate of social distancing of 2 m between individuals	7.48	8	1.97	7.62	8	1.53
	53. Non-contact meeting implementation rate	8.00	8	1.66	8.19	8	1.13
	54. Monitoring rate of facility workers or visitors	7.30	8	1.82	7.73	8	1.19
	55. Closing rate of public areas	7.22	8	1.79	7.54	8	1.14

### Round 2 – based on monitoring indicator evaluations

Similar to the first round of evaluation, an assessment of the same 55 indicators by monitoring domain was conducted in the second round (Table 2). For each indicator, the mean and median scores were obtained, which made a difference from the previous round. As with the results of the first round, two indicators were considered less important: “residential mobility rate” (6.85 points) and “high expenses for daily necessities experience rate” (6.77 points), which scored less than 7 points on average. However, in the open responses, only “residential mobility rate” was excluded from the final monitoring system since the importance of “high expenses for daily necessities experience rate” was emphasized. In addition, “restriction of public transportation” scored 7.04 points in the second round, highlighting its importance. Although the “restriction on domestic mobility” indicator was considered important in the first round (7.11 points), it was evaluated as 6.92 points in the second round. Both indicators were included in the direct policy level domain, which was excluded from the finalized monitoring system because the monitoring domain was modified based on the discussion of open responses. A detailed explanation is provided in the section discussing the open responses.

### Discussion about open-ended responses – monitoring domain

Most experts stated that the monitoring system domain was not clearly classified. The issue was raised that if monitoring system indicators are distinguished as being direct or indirect, some may overlap. Thus, it was suggested that the monitoring domains be delineated as ‘social distancing measures state, social distancing measures compliance, social distancing outcome’.

The indicators related to a state of national social distancing measures were included in the social distancing measures state. Unlike existing public health policies, Covid19-related policies have a short cycle of change. As the details of social distancing measures in South Korea are constantly changing, it is necessary to monitor changes of the measures.

The indicators affected by social distancing measures in action were included in the social distancing measures compliance. A key domain of the monitoring system is the ‘social distancing measures compliance’ domain. This domain consists of indicators that show how well people are being complied with social distancing.

Furthermore, the indicators related to the consequences of the social distancing measures and compliance (or behavior) in the social distancing outcome. This domain provides the information about the result of social distancing measures and compliance, and becomes evidence to control the level (details) of measures at the same time.

**Fig. 2:**
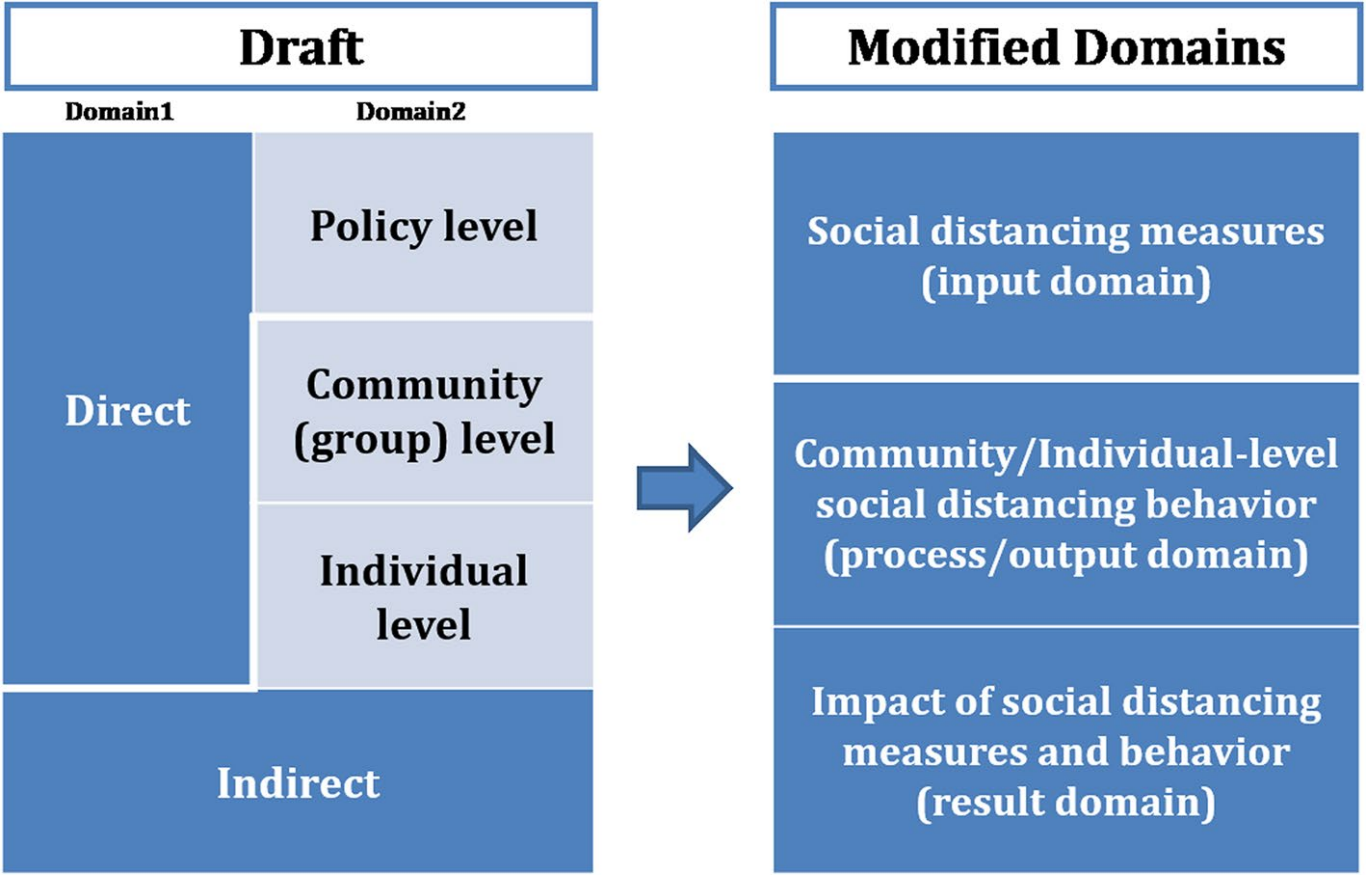
Modified monitoring domains based on the open-ended responses in the Delphi process

Therefore, the research team removed the direct/indirect level of domain 1. The research team revised the policy level of domain 2 to the “Social distancing measures state” domain, and the community/individual/indirect level of domain 2 to the “Social distancing measures compliance” domain. In addition, as the consequences of social distancing measures and behavior, the indicators for results were added. As such, the indicators were categorized as ‘social distancing measures state, social distancing measures compliance, social distancing outcome’ domains (Fig. 2).

### Discussion about open-ended responses – indicators

In addition to the index-specific evaluation scores, the necessary indicators were added and conventional indicators subdivided based on the experts’ feedback. Some indicators were combined or excluded according to the revised monitoring domains. According to the experts’ open response comments, 15 indicators were excluded, as these indicators overlapped with other indicators or had little relevance to social distancing. Instead, 23 new indicators were added.

#### Direct indicators: policy level → social distancing measures state

Nine monitoring indicators for direct indicators in the policy level domain were suggested by the team referring to the social distancing policies implemented worldwide. The experts provided their opinions about the nine monitoring indicators. Since the chosen indicators were based on the contexts of other countries, they have a limitation in that it is difficult to adequately monitor social distancing measures in Korea. Thus, indicators were changed to a state of social distancing measures in Korea. Therefore, measures such as the prohibition of events (e.g., general community meetings, sports events) and closure of institutions (e.g., multi-facilities, schools, large companies), and continuously emphasized personal quarantine guidelines were included as indicators in this domain.

#### Direct indicators: community level → social distancing measures compliance

Among the direct indicators presented by the researchers, four indicators were selected, displaying the degree of social distancing behavior at the community (group) level. The experts’ opinions were reflected as follows: Most experts suggested adding more indicators. “Whether to participate in online church worship services” and “the rate of conducting online lectures” (schools, private academic classes, etc.) were added based on representativeness and validation. There were recommendations to add “monitoring rate for employees or facility visitors,” “recording of guest book or QR code utilization rate,” and “temperature measurement rate of visitors,” which replaced the “facility measures performance rate” presented in the early version. Some suggested adding an “non-contact meeting conducting rate” indicator, but this was not reflected in the monitoring system since it was not possible to obtain data sources for the measurement.

#### Direct indicators: individual level → social distancing measures compliance

Among the direct indicators presented by the researchers, 38 displayed the degree of social distancing behavior at the individual level. The experts’ opinions were reflected as follows: It was suggested that a representative indicator be selected. Overall, the initial versions of the monitoring indicators and additional indicators based on the experts’ comments were collected to determine representative indicators (e.g., Since “Hand washing practice rate” encompasses “Use rate of hand sanitizers or soap”, “Hand washing practice rate” was selected as a representative indicator. For the same reason, “Facial mask wearing rate” was chosen instead of “Indoor facial mask wearing rate”.). Specifically, indicators such as “Change rate of movement at public transportation stops”, “residential mobility rate” and “purpose-specific transportation utilization rate” were excluded as monitoring indicators since they were not clearly defined because of data source limitations. In particular, the “residential mobility rate” was scored below 7 points in the evaluation.

For additional indicators, considering the definition and purpose of the process/output domain and indicator validity, indicators such as “rate of commuting with own car”, “Change rate of average travel distance”, “frequency of daily ventilation at home”, “Change rate of personal hygiene products” and “Perception of social distancing” were added. There were suggestions to add the measurement indicator “use of CCTV images and infrared sensors,” but this was difficult to reflect in the monitoring system because there are currently no available data sources.

#### Indirect indicators: indirect level → social distancing measures compliance

The indirect level of monitoring indicators suggested by the researchers displayed eight indicators that could not be considered directly affected by social distancing measures, but could be indirectly impacted. However, because of the vague domain definition of indirect effects, there were more opinions on domain modifications than opinions on indicators within the domain. In conclusion, all indicators within the indirect level were included in the domain of ‘Social distancing measures compliance’.

#### Social distancing outcome

Reflecting the opinion that the results of social distancing measures and compliance (or behavior changes) should be included in the monitoring system, this domain was added to the monitoring system. The 5 indicators related to controlling the stages of social distancing measures in Korea were added.

### Discussion about open-ended responses – data source

There were many negative opinions regarding the data sources. Most opinions revealed that currently available data sources have problems of timeliness, feasibility, and representativeness. However, these data sources were not excluded, as they were considered meaningful and could be immediately applicable to monitoring social distance policy. Thus, the monitoring system developed in this study was based on (1) the available data source at this stage and (2) data source accessible at the metropolitan level.

Finally, 12 data sources were included: 3 data sources with characteristics of primary data (Saramin survey, COVID-19 perception survey by Korea Research, Reports (current situation) by the National Disaster and Safety Status Control Center), and 9 data sources with secondary data (Google Mobility, Floating populations and phone calls provided by SKT in Tmap data, Floating populations data provided by KT, Credit card Sales data provided by Shinhan Card, Credit card Sales data provided by BC Card, Transportation data provided by Gyonggido Traffic control center, Korean Travel Survey, Korea Tourism Statistics, Air transport statistics). Table 3 provides details on each data source.

**Table 3:** Data source explanations

Types of data source	Name of data source	Details (explanations)
**Primary data sources**	Saramin survey	- Variables included: Type of business companies (large, mid-sized companies, small, etc.), type of industry (financial/insurance, information and IT), telecommuting or not, telecommuting reasons, stress due to social distancing, areas of stress due to social distancing, degree of feeling stress due to social distancing, need for social distancing, types of social distancing practice in daily life, perceptions regarding taking “3–4 days off if feeling sick,” reasons, applicability of measure, reasons why it cannot be implemented, experience commuting even when feeling sick, etc. - Data period: Once
	COVID-19 perception survey by Korea Research	- Variables included: Monthly, whether or not the following measures are in practice in regions (Seoul, Incheon/Gyeonggi, Daejeon/Sejong/Chungcheong, etc.): mask wearing, indoor mask wearing, use of hand sanitizer, daily life changes, restrictions on use of public transportation, restrictions on visits to multi-facilities, refraining from going or eating out, cancellation of gatherings (group dinner), control of work hours, cancellation or delay of hospital visits, etc. - Data period: Irregular
	Reports (current situation) by National Disaster and Safety Status Control Center	- Variables included: Number of business facilities such as Karaoke bars, Internet cafes, nightclubs, private educational institutes are under infectious disease preventive control management - Data period: Irregular
**Secondary data sources**	Google Mobility	- Variables included: Nationality, dates movement data for retail stores, leisure facilities, grocery stores, pharmacies, parks, public transportation stops, and working from home arrangements - Data period: Daily
	Floating population and phone calls provided by SKT (SK Telecom) in Tmap data	-Variables included: dates, Si-Gun-Gu, time (hour unit), sex, age (10-year), number of floating population, type of business classified as large scale (amenity, restaurant, medical facility), type of business classified as small scale, rate of making phone calls, search location ranks, etc. - Data period: Monthly
	Floating population data provided by KT	-Variables included: dates, Si-Gun-Gu, time (hour unit), sex, age (10-year), number of floating population - Data period: Monthly
	Credit card sales data provided by Shinhan Card	- Variables included: dates, Si-Gun-Gu, Eup-Myeon-Dong, type of business classified as large scale (restaurant, entertainment, travel, transportation), type of business classified as medium scale, type of business classified as small scale, sex, age, location, income level, credit card sales amount, number of credit card sales - Data period: Daily
	Credit card sales data provided by BC Card	- Variables included: dates, Si-Gun-Gu, Eup-Myeon-Dong, type of business classified as large scale (restaurant, entertainment, travel, transportation), type of business classified as middle scale, type of business classified as small scale, sex, age, location, income level, credit card sales amount, number of credit card sales - Data period: Daily
	Transportation data provided by Gyonggido Traffic control center	- Variables included: Si-Gun-Gu, month, day, number of passengers in express city bus/ regular bus/town bus, name of subway station, number of passengers using subway station, number of passengers in personal/corporate taxi, purpose-specific transportation use (commuting, going to school from home, working, shopping), sex, Si-Do, Si-Gun-Gu, purpose-specific transportation use by distance (units of 5 km) (commuting, going to school, shopping, leisure activities), type of transportation (car, bus, subway, taxi, bike, other) - Data period: Monthly/Annual
	Korean Travel Survey	- Variables included: Resident sex, age, occupation, education, number of people in household, household income, experience of domestic travel / resident or foreigner, number of visitors in major travel hotspot areas domestically, etc. - Data period: Monthly - Available data in 2018
	Korea Tourism Statistics	- Variables included: Purpose (tourism, commercial, public, study abroad, etc.), nationality of people arriving in Korea, number of departures of Korean residents, destination country, etc. - Data period: Monthly - Data source: Korea Tourism Organization
	Air transport statistics	- Variables included: International flights by region (Asia, Europe, North America, etc.), number of passengers, cargo weight in tons, number of domestic flights, number of passengers, cargo weight in tons, etc. - Data period: Monthly - Data source: Korea Airports Corporation, Incheon International Airport

### Final monitoring system

**Fig. 3:**
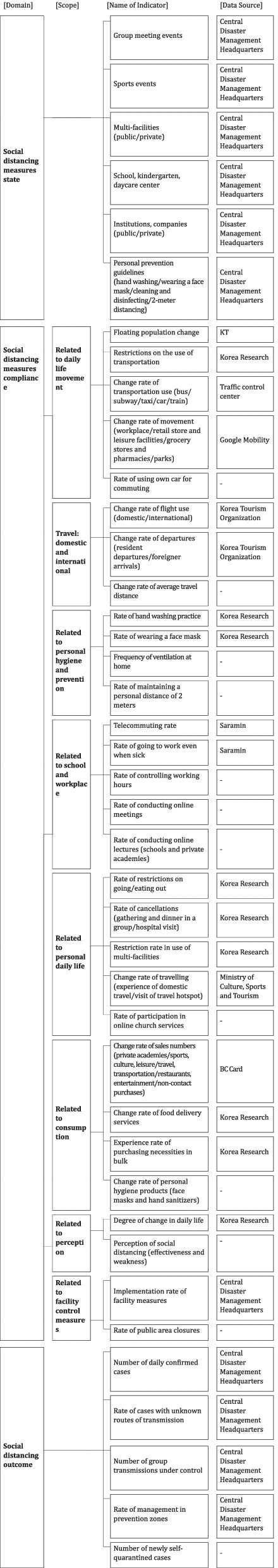
Final monitoring system

The final monitoring system was delineated as the ‘social distancing measures state, social distancing measures compliance, social distancing outcome’, and included 62 indicators (Fig. 3). Detailed definitions are provided in Additional file 1.

The social distancing measures state is a domain to check the status of changes in domestic social distancing measures. According to the social distancing stage in Korea, the measures taken for events (general gatherings, meetings, social events, and sports events) and in institutions (multi-facilities/schools/private companies), and continuously emphasized personal preventive practices were included as indicators. In other words, the indicators in this domain are the same as the measures of social distancing (not implementation or compliance).

The social distancing measures compliance is created during the process influenced by social distancing measures. This means we can see how well social distancing measures are being followed in this domain. This is a key domain of our monitoring system. It was divided into eight scopes including daily movement, domestic and international movement, personal hygiene and quarantine, school and workplace, personal daily life, consumption, risk perceptions, and facility measures, and included relevant indicators for each domain. In the daily movement scope, the rates for the change in floating population, restrictions on public transportation, change in types of transportation use, commuting, retail store and leisure facility movement, grocery store and pharmacy movement, movement in parks, and commuting with own car were included. In the national domestic and international movement scope, the change rates of the use of domestic flights, use of international flights, outbound travel by locals, arrival of foreigners, and average travel distance were included. In the personal hygiene and quarantine scope, the rates of hand washing practice, wearing of face masks, frequency of ventilation in the home, and practicing social distancing of 2 m between individuals were included. The school and workplace scope included the rates of implementation of telecommuting, experience of working even when sick, working hours adjustment, conducting online meetings, and online lectures. The personal and daily life scope included the rates of controlling one’s going out, cancellation of meetings, cancellation of hospital visits, restrictions on using multi-use facilities, domestic travel experience, changes in tourist visits to hotspots, and participation in online church worship services. The consumption scope included the change rates of private academy sales, number of sports/culture/leisure facilities sales, number of travel/transportation sales, number of restaurant/entertainment sales, non-contact purchases (home shopping, Internet shopping, etc.), food delivery, experience of purchasing necessities in bulk, and total sales of personal hygiene products (face masks, hand sanitizer). The (risk) perception scope included the degree of change in daily life and degree of perception of social distancing measures. Measures for facilities scope included the rates of implementation of these measures and closure of public areas.

The social distancing outcome is the consequence of social distancing measures and compliance (or behavior change) related to controlling the stages of these measures. Indicators such as number of daily confirmed cases, rate of unknown infection routes, status or number of mass outbreaks, rate of management in quarantine areas and number of new self-quarantines were included.

## Discussion

This study developed a social distancing monitoring system based on social distancing measures and available data about social distancing behavior in the country. The modified Delphi process was used to elicit the participation of experts from various fields such as infectious diseases, nursing, and health sciences. In total, 62 indicators were included in ‘social distancing measures state, social distancing measures compliance, social distancing outcome’ domains.

It is possible to create a social distancing monitoring system, by referring to the process and method of developing the monitoring system in this study. First, using national policy briefings and materials, list up social distancing measures. The list can be organized around measures that are taken on an ongoing basis. Second, identify available data to confirm compliance with measures or check whether measures are taken. The list is organized again based on available data, then a draft of monitoring system can be prepared. After that, the final monitoring system can be derived by refining the draft of monitoring system through Delphi process. And when referring to this study, the following must be considered.

In this study, a monitoring system was developed in consideration of Korea’s social distancing policy and environment. Since different countries have implemented social distancing policy in various ways, it is not appropriate to include their policy indicators (e.g., complete lockdowns, restrictions on movement between cities) that have not been considered in Korea. Even though similar policies may have been implemented in Korea, these are still difficult to apply because the recommended level or detailed methods may differ. For example, some countries implemented closure of educational institutions and others did not. Among countries that implemented closure of educational institutions, some countries have closed all schools, some countries have closed only kindergartens∙primary∙secondary schools, and some countries have implemented part-time school attendance. In addition to school closures, there are countless social distancing measures, and the indicators to be checked also vary depending on whether or not the measures are taken and the intensity of the measures. Thus, the social distancing monitoring system should be developed by reflecting the environment and policies implemented in each country.

The domain-specific suggestions of the monitoring system presented in this study are as follows: Currently, the social distancing measures state simply monitors changes of the social distancing measure status. In the future, a policy evaluation study is needed to evaluate the feasibility and appropriateness of domestic social distancing stages. Furthermore, it will be necessary to develop tools to compare the levels of social distancing policies in Korea and other countries. For the social distancing measures compliance, a large number of indicators were provided by the surveys. However, various national monitoring methods are still needed to measure practice rates in a “realistic” or “objective” way, such as using CCTV to monitor hand washing and the wearing of masks, attaching infrared sensors on faucets, conducting observation surveys, and using video conference equipment in public conference rooms. In addition, it is necessary to develop and support measurement methods for indicators that cannot be added because of the lack of proper data sources. The social distancing outcome should clarify the definition and formula of the result indicators of COVID-19, which is intermittently produced by the National Disaster and Safety Status Control Center, so that the actual effectiveness of social distancing policy can be accurately determined.

Various types of environmental and institutional support must be presented for the monitoring systems developed in this study to work realistically. Above all, a national social distancing monitoring system should be established that combines all policies from each government department. It is necessary to design systematic surveys to assess the effectiveness of social distancing behavior in a more objective way, rather than over the long term. And a clear definition of social distancing measures is needed. The current social distancing policies in Korea include national/institutional-level measures (e.g., business restrictions on high-risk facilities, and school regulations, etc.) and compliance with individual-level quarantine guidelines. While policies have been constantly changing, no clear definition has been provided. So, confusion in the community continues in line with changing guidelines, as the definition of social distancing policy is not clear [27, 28]. Therefore, it is essential to draw social consensus on the definition of social distancing policies, and share them in a more consistent way.

Although the vaccine for COVID-19 has been developed and used, it has been announced that it will still take a long time to achieve the desired expectations [3, 4]. Social distancing measures to reduce the transmission of COVID-19 are being implemented worldwide, but research is lacking on how the policy affects the prevalence and mortality of COVID-19 [29]. It is very important to assess and monitor the effectiveness of social distancing policy not only for COVID-19 but also for an effective response to possible new infectious diseases in the future [29].

### Strengths and weaknesses

The limitations of this study are as follows: First, a guidance on using the framework as a tracking tool is not provided in this study. Second, this study developed monitoring indicators based on currently available data sources, excluding infinite measurement methods and indicators that can measure social distancing behavior. Third, clinical healthcare professionals related to COVID-19 and healthcare system development and analysis specialists were only invited to participate in the modified Delphi process. In Korea, clinical healthcare professionals are the main discussion group (or the policy decision-making group) for all public health measures, including social distancing measures. It is a characteristic feature of the public health system in Korea.

Nevertheless, this study is meaningful in that it developed a comprehensive monitoring system for compliance of social distancing for the first time in Korea and provided available data sources by indicator. In addition, based on the social and economic impact created by social distancing [6], we developed indicators that distinguish domains by field and quantitatively understand the degree of social distancing practice in each. Since the COVID-19 pandemic is prolonged, not only in Korea but also in other countries, we can refer to our developed monitoring system for decision-making regarding the timing and intensity of various infectious disease response policies.

## Conclusion

For the first time in Korea, this study developed a comprehensive and realistic monitoring system for checking if social distancing measures are being followed well by providing data sources that can be immediately identified by indicators. This study will be available not only in Korea but also in other countries that need to develop monitoring systems for social distancing. However, the results of this study should be applied in accordance with each country’s measures and contexts (environments), and the status continuously reported, analyzed, and updated, even after the monitoring system is established. Subsequently, efforts are required to continuously monitor the status of the system and utilize it to improve social distancing measures.

## Supplementary Information

**Additional file 1.**

## Data Availability

The datasets used and/or analysed during the current study are available from the corresponding author on reasonable request.

## Abbreviations

WHO: World health organization
ECDC: European centre for display prevention and control
