# Supplementary material for: Development of a social distancing monitoring system in Republic of Korea: results of a modified Delphi process
Source: BMC Public Health. 2022 Apr 29;22:860. doi: 10.1186/s12889-022-13277-8 (PMC9053561; doi:10.1186/s12889-022-13277-8)
Supplement: Supplementary file 1 — Additional file 1. [file 12889_2022_13277_MOESM1_ESM.docx]

**Social distancing measures(Input domain) indicators:** These indicators can be modified due to policy changes (based on measures in Korea as of 2021.01.01)

| **Category** | **Level 1** | **Level1.5** | **Level 2** | **Level 2.5** | **Level 3** |
| --- | --- | --- | --- | --- | --- |
|  | **Everyday quarantine** | **Regional spread** | | **Nationwide pandemic** | |
| Facilities in priority control | Restriction on number of people entering in etc. Mandatory to follow Core prevention measures | Reinforcement on restrictions of Number of people entering in, Ban in high-risk activities | Prohibition of Gathering in 5 major types of Entertainment facilities | Prohibition of gather in Door-to-door sales (direct sales promotion center), Karaoke, indoor concert hall | All facilities are on restrictions |
|  |  |  | For other facilities: Closed or reinforced restrictions after 9 pm, if violated, complete suspension |  |  |
| Facilities in general control | Normally operated but should follow basic 3 quarantine measures in mandatory | By facility features, restriction on number of people use | Reinforcement on number of people use the facility, ban in high-risk activities | Closed or reinforced restrictions after 9 pm, if violated, complete suspension |  |
| Other facilities | Normal operation | | Wearing fascial masks in mandatory | Restriction number of people using the facility |  |
| National/public facilities | Limiting to 50% of people in bicycle racing, horse racing etc. | Limiting to 20%of people in bicycle racing, horse racing etc., Limiting to 50% of people in other facilities | Suspension of bicycle racing, horse racing etc., Limiting to 30%of people using other facilities | Suspension of sports facilities, bicycle racing, horse racing etc., Limiting to 30% of people using other facilities | All facilities are suspended |
| Social welfare facilities (including day care center) | Operation under strict quarantine control | | | | Recommended to close, emergency care service is maintained |
|  |  | | | |  |
|  | * If necessary, some facilities will be closed and only necessary services such as emergency care are provided, considering the spread of infections in epidemic areas, risks by facilities, and quarantine management conditions. | | | |  |
| Wearing masks in mandatory | Facilities (in priority control and general control), public transportation, hospitals, pharmacies, nursing homes, day and night shelters, rally and demonstration places, indoor sports stadium, high-risk workplace etc. | Places in level 1 and outdoor sports stadium | All indoor areas, high-risk outdoor activities | All indoor areas, areas with impossible to maintain distancing in 2meters | |
| Social gatherings and events | Events with more than 500 people need to be reported and consulted by local governments, should follow quarantine measures in mandatory | Level 1 measures are maintained, some events, such as festivals, are prohibited from more than 100 people | Prohibition of more than 100 people | Prohibition of more than 50 people | Prohibition of more than 10 people |
| Spectators of sports games | 50% of spectators can enter | 30% of spectators can enter | 10% of spectators can enter | No spectators | Banned |
| Public transportation use | Wearing fascial masks in mandatory | | No eating inside of transportation (exception for international flights) | Limiting to ticket within 50% (KTX, express bus etc.) (except for flight tickets) | Limiting to ticket within 50% (KTX, express bus etc.) (except for flight tickets) |
| Schools | Density of 2/3 rule, changeable | Density of 2/3 should be followed | Density of 1/3 rule (high school: 2/3) can open within density of 2/3 | Density of 1/3 should be followed | Distance learning |
| Religious services | ※ able to determine details and targets of specific measures in consultation with the religious community in accordance with prevention and group transmission of viruses situations during stage adjustment | | | | |
|  | Take a seat in leaving a seat in between, refrain from meeting and eating (ban on overnight event) | Restriction in number of seats to 30% for regular worship services, ban on meeting and eating | Restriction in number of seats to 20% for regular worship services, ban on meeting and eating | Untact service, Restriction in number of participants to 20 for regular worship services, ban on meeting and eating | Only 1 person video is allowed, ban on meeting and eating |
| Workplaces | Recommendation for telecommuting at an appropriate rate by institution and department (for example, at 1/5 ratio) | Recommendation for expanding telecommuting by institution and department (for example, at 1/3 ratio) | | Recommendation for telecommuting more than 1/3 of the population in the workplace | Telecommuting in mandatory other than required workers |
|  | Wearing masks in mandatory for high-risk workplaces | Mandatory of wearing masks in high-risk workplace, ventilation and hygiene, distancing between workers etc.양식의 맨 아래 | | | |

*Above indicators can be modified due to policy changes (based on measures in Korea as of 2021.01.01)

**Community/Individual-level social distancing behavior(Process/output domain) indicators**

| **Name of Indicator** | **Definition** | | |
| --- | --- | --- | --- |
| Floating population change | Definition | | **Proportion of floating population** |
|  | Numerator | | Number of floating populations in same period of year 2020 in specific region *100 |
|  | Denominator | | Number of floating populations in same period of year 2019 in specific region |
| Restrictions on the use of transportation | Definition | | **Degree of restriction of using public transportation since COVID-19 pandemic** |
|  | Numerator | | Number of people who have not used public transportation since COVID-19 pandemic *100 |
|  | Denominator | | All respondents |
| Change rate of transportation use (bus, subway, taxi, car, train) | Definition | | **percentage of changes in public transportation usage in 2020 compared to the previous year** |
|  | Numerator | | Frequency use of public transportation (bus, subway, taxi, car, railways) in same period of 2020 *100 |
|  | Denominator | | Frequency use of public transportation (bus, subway, taxi, car, railways) in same period of 2019 |
| Change rate of movement (workplace) | Definition | | **Percentage of change in workplace movement as of 2020 compared to the baseline value** |
|  | Numerator | | Movement in workplace in 2020 (present) |
|  | Denominator | | Movement in workplace for 5 weeks (baseline), Jan. 3^rd^, 2020 to Feb. 6^th^, 2020 |
| Change rate of movement (retail store, leisure facilities) | Definition | | **Percentage of movement changes in restaurants, cafes, shopping centers, amusement parks, museums, libraries, and theaters as of 2020 compared to the baseline value** |
|  | Numerator | | Movement in restaurants, cafes, shopping centers, amusement parks, museums, libraries, and theaters in 2020 (present) |
|  | Denominator | | Movement in restaurants, cafes, shopping centers, amusement parks, museums, libraries, and theaters for 5 weeks (baseline), Jan. 3^rd^, 2020 to Feb. 6^th^, 2020 |
| Change rate of movement (grocery stores, pharmacies) | Definition | | **Percentage of movement changes in places such as grocery stores, food retail stores, agricultural markets, specialty food stores, drugstores, and pharmacies as of 2020 compared to the baseline value** |
|  | Numerator | | Movement in places such as grocery stores, food retail stores, agricultural markets, specialty food stores, drugstores, and pharmacies in 2020 (present) |
|  | Denominator | | Movement in places such as grocery stores, food retail stores, agricultural markets, specialty food stores, drugstores, and pharmacies for 5 weeks (baseline), Jan. 3^rd^, 2020 to Feb. 6^th^, 2020 |
| Change rate of movement (parks) | Definition | | **Percentage of movement changes in places such as** **national parks, public beaches, berths, pet parks, plazas, and public gardens as of 2020 compared to the baseline value** |
|  | Numerator | | Movement in places such as national parks, public beaches, berths, pet parks, plazas, and public gardens in 2020 (present) |
|  | Denominator | | Movement in places such as national parks, public beaches, berths, pet parks, plazas, and public gardens for 5 weeks (baseline), Jan. 3^rd^, 2020 to Feb. 6^th^, 2020 |
| Rate of using own car for commuting | Indicator to be developed | | |
| Change rate of flight use (domestic) | Definition | | ***Rate of changes in domestic flights in 2020 compared to the previous year*** |
|  | Numerator | | Number of people using domestic flights within the same period in 2020 *100 |
|  | Denominator | | Number of people using domestic flights within the same period in 2019 |
| Change rate of flight use (international) | Definition | | **rate of change in international flights in 2020 compared to the previous year** |
|  | Numerator | | Number of people using international flights within the same period in 2020 *100 |
|  | Denominator | | Number of people using international flights within the same period in 2019 |
| Change rate of departures (resident departures) | Definition | | **Rate of change in departure of Koreans in 2020 compared to the previous year** |
|  | Numerator | | Number of Koreans who left the country during the same period in 2020 *100 |
|  | Denominator | | Number of Koreans who left the country during the same period in 2019 |
| Change rate of departures (foreigner arrivals) | Definition | | **Rate of change in foreigner arrivals in 2020 compared to the previous year** |
|  | Numerator | | Number of foreigners entering in country during the same period in 2020 *100 |
|  | Denominator | | Number of foreigners entering in country during the same period in 2019 |
| Change rate of average travel distance | Indicator to be developed | | |
| Rate of hand washing practice | Definition | | Percentage of hand washing people |
|  | Numerator | | Number of people who washed hands*100 |
|  | Denominator | | All respondents |
| Rate of wearing a face mask | Definition | | **Percentage of people who wear masks outdoors or indoors** |
|  | Numerator | | Number of people who wore fascial masks (outdoors/indoors) *100 |
|  | Denominator | | All respondents |
| Frequency of ventilation at home | Indicator to be developed | | |
| Rate of maintaining a personal distance of 2 meters | Indicator to be developed | | |
| Telecommuting rate | Definition | | **rate at which a company's telecommuting plan exists or is in place** |
|  | Numerator | | Number of companies that are willing to carry out telecommuting (implementing planned) *100 |
|  | Denominator | | Number of responded companies |
| Rate of going to work even when sick | Definition | | **Percentage of work experience even if felt sickness** |
|  | Numerator | | number of people who responded that they had endured to going work even if felt sickness. *100 |
|  | Denominator | | All respondents |
| Rate of controlling working hours | Indicator to be developed | | |
| Rate of conducting online meetings | Indicator to be developed | | |
| Rate of conducting online lectures (schools and private academies) | Indicator to be developed | | |
| Rate of restrictions on going/eating out | Definition | **restriction rate of going out/ eating out** | |
|  | Numerator | Number of people who have responded that they have refrained from going out/out since COVID-19 outbreak *100 | |
|  | Denominator | All respondents | |
| Rate of cancellations (gathering, dinner in a group) | Definition | **degree of cancellation of meetings and dinners since COVID-19 outbreak** | |
|  | Numerator | Number of people who have canceled meetings and dinners since COVID-19 outbreak *100 | |
|  | Denominator | All respondents | |
| Rate of cancellations (hospital visit) | Definition | **Degree of cancellation of hospital visits since COVID-19 outbreak** | |
|  | Numerator | number of people who responded that they postponed or canceled their scheduled visit to the hospital after COVID-19 outbreak*100 | |
|  | Denominator | All respondents | |
| Restriction rate in use of multi-facilities | Definition | **Degree of refrain from entering in theaters, department stores, large discount stores, etc. after COVID-19 outbreak.** | |
|  | Numerator | Number of people who responded that they have refrained from entering in department stores, large discount stores, etc. since COVID-19 outbreak. *100 | |
|  | Denominator | All respondents | |
| Change rate of travelling (experience of domestic travel) | Definition | **rate of change in domestic travel experience in 2020 compared to the previous year** | |
|  | Numerator | Experience domestic travel during the same period in 2020 *100 | |
|  | Denominator | Experience domestic travel during the same period in 2019 | |
| Change rate of travelling (visit of travel hotspot) | Definition | **rate of change of visitors to tourist attractions (hotspots) in 2020 compared to the previous year** | |
|  | Numerator | Number of tourist attractions (hotspots) in the same period of 2020 *100 | |
|  | Denominator | Number of tourist attractions (hotspots) in the same period of 2019 | |
| Rate of participation in online church services | Indicator to be developed | | |
| Change rate of sales numbers (private academies ) | Definition | **percentage of changes in the number of private academy sales in 2020 compared to the previous year** | |
|  | Numerator | Number of private academy sales in the same period in 2020 *100 | |
|  | Denominator | Number of private academy sales in the same period in 2019 | |
| Change rate of sales numbers (sports, culture, leisure) | Definition | **percentage of changes in the number of sports/culture/leisure facilities sales in 2020 compared to the previous year** | |
|  | Numerator | Number of sports/culture/leisure facilities sales in the same period in 2020 *100 | |
|  | Denominator | Number of sports/culture/leisure facilities sales in the same period in 2019 | |
| Change rate of sales numbers (travel, transportation) | Definition | **percentage of changes in the number of travel/transportation sales in 2020 compared to the previous year** | |
|  | Numerator | Number of travel/transportation sales in the same period in 2020 *100 | |
|  | Denominator | Number of travel/transportation sales in the same period in 2019 | |
| Change rate of sales numbers (restaurants, entertainment) | Definition | **percentage of changes in the number of restaurants/entertainment sales in 2020 compared to the previous year** | |
|  | Numerator | Number of restaurants/entertainment sales in the same period in 2020 *100 | |
|  | Denominator | Number of restaurants/entertainment sales in the same period in 2019 | |
| Change rate of sales numbers (non-contact purchases) | Definition | **degree of use of home shopping and internet shopping after COVID-19 outbreak** | |
|  | Numerator | number of people who responded that home shopping and Internet shopping have increased since COVID-19 outbreak*100 | |
|  | Denominator | All respondents | |
| Change rate of food delivery services | Definition | **Degree of using food delivery services after COVID-19 outbreak** | |
|  | Numerator | Number of people who have responded to increased food order delivery since COVID-19 outbreak. *100 | |
|  | Denominator | All respondents | |
| Experience rate of purchasing necessities in bulk | Definition | **degree of mass purchase of daily necessities since COVID-19 outbreak** | |
|  | Numerator | number of people who responded that they have purchased more daily necessities than usual such as instant noodles and instant rice since COVID-19 outbreak. *100 | |
|  | Denominator | All respondents | |
| Change rate of personal hygiene products (face masks/hand sanitizers) | Indicator to be developed | | |
| Degree of change in daily life | Definition | **Change of daily life since COVID-19 pandemic** | |
|  | 0 score : no daily life after COVID-19 pandemic  100 score: same daily life as before and after COVID-19 pandemic | | |
| Perception of social distancing (effectiveness/weakness) | Indicator to be developed | | |
| Implementation rate of facility measures | Definition | **Implementation rate of infection prevention measures in high-risk facilities (Karaoke, Internet cafe, nightclubs, private academy, teaching center, etc.)** | |
|  | Numerator | Number of facilities that have implemented infection prevention measures *100 | |
|  | Denominator | Number of facilities for inspection | |
| Rate of public area closures | Indicator to be developed | | |

**Impact of social distancing measures and behavior(Result domain) indicators**

| Name of indicator | Definition | |
| --- | --- | --- |
| Number of daily confirmed cases | Definition | A person who has been confirmed to be infected with an infectious disease pathogen in accordance with the inspection criteria for diagnosis regardless of clinical conditions |
| Rate of cases with unknown routes of transmission | Definition | Percentage of confirmed patients whose transmission route is unknown |
| Number of group transmissions under control | Definition | Number of group transmission under management |
| Rate of management in prevention zones | Definition | Percentage of confirmed cases among self-quarantine cases |
| Number of newly self-quarantined cases | Definition | Number of people who have been issued a self-quarantine notice from the public health center in charge of the actual residence |
